# “It is not the diet; it is the mental part we need help with.” A multilevel analysis of psychological, emotional, and social well-being in obesity

**DOI:** 10.1080/17482631.2017.1306421

**Published:** 2017-04-18

**Authors:** Kathryn Rand, Michael Vallis, Megan Aston, Sheri Price, Helena Piccinini-Vallis, Laurene Rehman, Sara F.L. Kirk

**Affiliations:** ^a^ Healthy Populations Institute, Dalhousie University, Halifax, Canada; ^b^ Department of Psychiatry, Dalhousie University, Halifax, Canada; ^c^ School of Nursing, Dalhousie University, Halifax, Canada; ^d^ Department of Family Medicine, Dalhousie University, Halifax, Canada; ^e^ School of Health and Human Performance, Dalhousie University, Halifax, Canada

**Keywords:** Obesity, well-being, mental well-being, weight bias, Socio-Ecological Model

## Abstract

In this research, we explored the psychological, emotional, and social experiences of individuals living with obesity, and perceptions of health care providers. We conducted a theoretical thematic analysis using two theoretical frameworks applied to transcripts from a previous qualitative study. Themes from a mental well-being framework were subsequently categorized under five environmental levels of the Social-Ecological Model (SEM). Key mental well-being themes appeared across all levels of the SEM, except the policy level. For the individual environment, one main theme was food as a coping mechanism and source of emotional distress. In the interpersonal environment, two themes were (a) blame and shame by family members and friends because of their weight and (b) condemnation and lack of support from health professionals. In the organizational environment, one main theme was inadequate support for mental well-being issues in obesity management programmes. In the community environment, one major theme the negative mental well-being impact of the social stigma of obesity. An overarching theme of weight stigma and bias further shaped the predominant themes in each level of the SEM. Addressing weight stigma and bias, and promoting positive mental well-being are two important areas of focus for supportive management of individuals living with obesity.

## Introduction

Many weight-management programmes have had limited long-term success (Curioni & Lourenco, 2005). Research suggests that the healthcare system and professionals who work in it struggle to provide the level or type of support that individuals living with obesity feel that they need for effective weight management (Kirk et al., 2014). This suggests a need for a greater focus on quality of life factors in weight-management programmes, such as psychological, emotional, and social well-being (Leske, Strodl, & Hou, 2012; Vallis, 2016). These together represent three core components of mental well-being (Aggarwal, 2011).

Psychological well-being includes self-acceptance, autonomy, environmental mastery, purpose in life, and personal growth (Ryff, 1989). Research indicates that the psychological well-being of those living with obesity may be compromised due to “distress over obesity,” defined as “the degree to which an individual is concerned and unhappy about his or her body and the impact of excess weight” (Vallis, 2016, p. 197; see also Butler et al., 1999; Wee, Davis, Huskey, Jones, & Hamel, 2012). Many individuals living with obesity experience this distress because they blame themselves for having excess weight, especially if they have tried to lose weight in the past and failed to achieve their desired weight or shape (Duloo & Montani, 2015; Gatineau & Dent, 2011). Distress over obesity is heightened when people perceive themselves to have poorer health because of obesity-related conditions such as chronic pain, osteoarthritis, and cardiovascular disease (Taylor, Forhan, Vigod, Mcintyre, & Morrison, 2013).

Emotional well-being encompasses emotions associated with positive and negative affect, happiness, and life satisfaction (Simsek, 2011; Snyder & Lopez, 2007; Watson, Clark, & Tellegen, 1988). The emotional well-being of those living with obesity may be negatively influenced by eating patterns that develop from widespread availability of palatable foods as well as increased psychological availability of food through a change in social norms that make it acceptable and desirable to partake in eating whenever and wherever available (Lowe & Butryn, 2007). Hedonic hunger is a dimension of appetite and food intake that includes frequent thoughts, feelings, and urges about consuming palatable foods in the absence of prolonged food deprivation (Lowe & Butryn, 2007). Those who struggle with hedonic hunger report feelings of guilt, shame, and fear over their perceived lack of control over food intake and failed weight loss attempts (Berridge, Ho, Richard, & Difeliceantonio, 2010; Gatineau & Dent, 2011).

Social well-being comprises social integration, acceptance, contribution, actualization, and coherence (Keyes, 1998). The social well-being of those living with obesity may be negatively affected due to the social stigma of obesity. Pervasive negative weight-related attitudes in society, such as that those living with obesity are lazy and unintelligent, may result in discriminatory behaviours in the workplace and in healthcare settings, and social opportunities (Puhl & Heuer, 2009; Taylor et al., 2013). Social stigma, in turn, influences both emotional and psychological well-being through the internalization of anti-obesity attitudes. Anti-obesity attitudes create distress and shame for those living with obesity, lowering their self-esteem and sense of self-worth (Taylor et al., 2013; Vallis, 2016).

Taken together, psychological, emotional, and social well-being can be conceptualized as core components of overall mental well-being (Aggarwal, 2011). Mental well-being can, in turn, be defined as “a state of well-being in which every individual realizes his or her own potential, can cope with the normal stresses of life, can work productively and fruitfully, and is able to contribute to her or his community” (World Health Organization [WHO], 2005). However, mental well-being is not only a product of an individual’s biology and genes, but is also determined by socio-ecological factors, such as interactions in one’s family, communities, organizations, and society (WHO, 2005). A socio-ecological approach, that considers how individual-, interpersonal-, organizational-, community-, and policy-level environments might impact individual behaviours, is being increasingly used in health promotion research because it addresses the importance of both individual and environmental influences on health (Mcleroy, Bibeau, Steckler, & Glanz, 1988).

There is currently a gap in qualitative research around the lived experience of obesity in relation to mental well-being, particularly from a socio-ecological perspective (Mcleroy et al., 1988). We aimed to address this gap by exploring how individuals living with obesity, and healthcare providers (HCPs) that work with people living with obesity, understand the influence of psychological, emotional, and social well-being across these multiple spheres of influence. Specifically, we sought to answer the following questions: How do those living with obesity describe their experiences with psychological, emotional, and social issues? What supports do those living with obesity feel they need to promote positive mental well-being? What psychological, emotional, and social issues do HCPs perceive are problems for their patients living with obesity? What supports do healthcare professionals provide to promote the positive mental well-being of their patients?

## Methods

In 2010–2011, we conducted qualitative semi-structured interviews in an eastern Canadian province with individuals who self-identified with obesity, with HCPs, and with policymakers. The original research was conducted using feminist poststructuralism (Butler, 1992; Cheek, 2000; Foucault, 1983; Scott, 1992) as the guiding methodology. Feminist poststructuralism focuses on understanding how personal experiences are constructed through social and institutional discourses through relations of power (Aston, Price, Penney, & Kirk, 2011). Three cross-cutting themes were identified across the groups of participants, including aspects of blame relating to obesity development, tensions between obesity management and prevention, and a prevailing discourse that was not supportive to the management of obesity (Kirk et al., 2014). These themes are described in detail elsewhere (Kirk et al., 2014). In addition, among individuals with obesity and HCPs, psychological and emotional issues were noted as being important aspects in obesity management, thereby providing the rationale for this secondary data analysis.

Semi-structured interview guides developed in the original study explored the perceptions and experiences of individuals living with obesity and their interactions with the healthcare system, as well as how HCPs perceived weight management in the healthcare system (Kirk et al., 2014). In Table I, we provide examples of overarching questions included for individuals with obesity and HCPs, who are the focus of this secondary data analysis. There were no specific questions or probes during the interview process on the theme of mental well-being; therefore, all responses were non-solicited reactions to lived experiences with excess body weight or with supporting clients living with obesity.

**Table I.** Sample interview questions according to participant groups.

Individuals living with obesity	Healthcare providers
Can you tell me what brought you here to participate in our study?	Can you talk about your overall experience in providing care to overweight and obese clients?
Can you talk about your overall experience regarding your weight?	What do you talk about with overweight and obese clients?
Can you tell me about your overall experience discussing your weight with healthcare professionals?	Can you talk about your experience in delivering care to overweight and obese clients?

Kirk et al. (2014, p. 792).

Ethical approvals for the original study were received both at the institutional level and within each health district that participants were recruited from. Recruitment of participants was completed through a variety of media and print advertisements as well as targeted emails to professional organizations. The recruitment of individuals living with obesity and HCPs was completed independently with no match-up of patient to practitioner. Written informed consent was obtained by all participants following the distribution of the study information package, prior to the in-person interviews. A trained research coordinator and assistant conducted the face-to-face interviews, which were audio taped and transcribed verbatim. All interviews were coded by number to maintain confidentiality. Data collection ended when saturation was reached, as indicated by no new themes or codes being identified in additional participant transcripts, and the study methodology had sufficient detail to allow for replication of findings (Patton, 2002). Complete details of the methodology used and findings from the original study are available elsewhere (Aston et al., 2011; Kirk et al., 2014; Price, Aston, Rehman, & Kirk, 2015).

### Participants

Secondary data analysis comprised interview transcripts from 19 individuals living with obesity, and 16 primary HCPs (eight dietitians, four family physicians, and four nurses). Anyone who identified as having experience living with obesity within the province, as well as HCPs with experience working with clients living with obesity, was eligible to participate in the original study. Most participants were female, with fewer than a quarter reported as being male. The dominance of the female gender represented in the HCPs reflects the predominance of the female gender within two of the three professions, i.e., nursing and dietetics. The dominance of the female gender in individuals living with obesity may be because females tend to seek out medical help more than their male counterparts do.

### Theoretical frameworks

Two theoretical frameworks were selected to guide this secondary data analysis. First, a mental well-being framework was developed out of the WHO (2005) concept that complete mental well-being is an essential aspect of achieving health. It represents what the WHO (2005) would define as the main mental well-being domains of psychological, emotional, and social well-being (see Table II). We applied this framework by comparing the dimensions of emotional, psychological, and social well-being as outlined in Tables III, IV, V to participant narratives, to analyse which aspect(s) of mental well-being were influenced by their experiences with obesity.

**Table II.** Emotional, psychological, and social domains of mental well-being.

Emotional well-being dimensions	Psychological well-being dimensions	Social well-being dimensions
Positive affect	Self-acceptance	Social integration
Negative affect	Positive relations with others	Social acceptance
Happiness	Autonomy	Social contribution
Life satisfaction	Environmental mastery	Social actualization
	Purpose in life	Social coherence
	Personal growth	

Keyes (1998, pp. 122–123); Ryff (1989, p. 1071); Simsek (2011); Watson et al. (1988, p. 1070).

**Table III.** Emotional well-being domain of mental well-being.

Emotional well-being dimensions	Emotion examples below were noted in participant dialogues to determine an overall positive or negative affect.
Positive affect	Interested, excited, strong, enthusiastic, proud, alert, inspired, determined, attentive, active
Negative affect	Distressed, upset, guilty, scared, hostile, irritable, ashamed, jittery, afraid
Happiness	Having a general feeling and experience of pleasure, contentment, and joy
Life satisfaction	A sense of contentment and peace stemming from small gaps between wants and needs

Simsek (2011); Snyder & Lopez (2007, pp. 61, 67); Watson et al. (1988, p. 1070).

Second, the Social-Ecological Model (SEM) was applied to categorize the mental well-being themes identified. The SEM is a theory-based framework used to understand how individual behaviour is influenced by and influences the environments of the social system (Bronfenbrenner, 1977). The SEM includes five different environmental levels that influence human behaviour: the individual, interpersonal, organizational, community, and policy levels. Once the mental well-being themes were identified within participant transcripts, these themes were categorized under the appropriate levels within the SEM framework as outlined in Table VI. Use of the SEM framework within this specific research determined where individuals living with obesity are facing experiences that adversely affect their well-being. Identifying what aspects of their environments are affected by psychological, emotional, and social issues is useful to direct future research on where to implement support strategies that promote positive mental well-being for those living with obesity.

**Table IV.** Psychological well-being domain of mental well-being.

Psychological well-being dimensions	Definitions
Self-acceptance	“The individual’s sense of self-acceptance.”
Autonomy	“Self-determination, independence, and the regulation of behavior from within.”
Environmental mastery	“The individual’s ability to choose or create environments suitable to his or her psychic conditions is deemed as a characteristic of mental well-being. ”
Purpose in life	“Mental well-being is deemed to include beliefs that give one the feeling there is purpose in and meaning to life. The definition of maturity also emphasizes a clear comprehension of life’s purpose, a sense of directedness, and intentionality.”
Personal growth	“Optimal psychological functioning requires not only that one achieve the prior characteristics, but also that one continue to develop one’s potential, to grow and expand as a person. The need to actualize oneself and realize one’s potentialities is central to the clinical perspectives on personal growth.”

Ryff (1989, p. 1071).

**Table V.** Social well-being domain of mental well-being.

Social well-being dimensions	Definitions
Social integration	“The evaluation of the quality of one’s relationship to society and community. Healthy individuals feel that they are a part of society.”
Social acceptance	“The construal of society through the character and qualities of other people as a generalized category. Individuals who illustrate social acceptance trust others, think that others are capable of kindness, and believe that people can be industrious.”
Social contribution	“The evaluation of one’s social value. It includes the belief that one is a vital member of society, with something of value to give to the world.”
Social actualization	“The evaluation of the potential and the trajectory of society. This is the belief in the evolution of society and the sense that society has potential which is being realized through its institutions and citizens. Healthier people are hopeful about the condition and future of society, and they can recognize society’s potential.”
Social coherence	“The perception of the quality, organization, and operation of the social world and it includes a concern for knowing about the world. Healthier people not only care about the kind of world in which they live, but also feel that they can understand what is happening around them.”

Keyes (1998, pp. 122–123).

**Table VI.** Exploring mental well-being using the Social-Ecological Model.

Level of the Social-Ecological Model	Connection to mental well-being
Individual	On this level, it is important to reflect on the individuals’ own perceptions of themselves in relation to their obesity and how it affects their mental well-being.
Interpersonal	On this level, it is important to investigate the participants’ relationships with family members, friends, and co-workers and how this influences their mental well-being.
Organizational	On this level, it is important to investigate the participants’ experience with the healthcare system in relation to their mental well-being concerns.
Community	On this level, it is important to look at the broader community’s relationship with those living with obesity and how this has affected their mental well-being.
Policy	On this level, it is important to look at the participants’ awareness of policies in place for the provision of mental well-being support, if any.

Bronfenbrenner (1977).

### Data analysis

We conducted a secondary theoretical thematic analysis using the six steps as outlined by Braun and Clarke (2013). This included (1) *familiarizing yourself with the data*, whereby transcripts were read in their entirety, multiple times, in order to become familiar with the whole text before any division into pertinent parts; (2) *coding*, whereby notes in the margins of the text identified emerging concepts then organized according to the aim of the study; (3) *searching for themes*, whereby codes within the data were used to construct prominent themes and collate all coded data into each relevant theme; (4) *reviewing themes*, whereby the full data set was reviewed to confirm the themes were relevant to the research purpose and told a compelling story about the data; (5) *defining themes*, whereby an analysis of the themes developed a concise and informative name for each theme; and, lastly, (6) *writing up*, whereby a coherent story about the data was developed through choosing rich data extracts pertinent to each theme and contextualizing it in relation to existing literature.

## Results

Key themes influencing psychological, emotional, and social well-being were identified within the individual, interpersonal, organizational, and community levels of the SEM and across participant groups, as follows.

### Individual level

Within the individual level of the SEM, one theme was identified, which was *food as a coping mechanism and source of emotional distress*. The use of food as a coping mechanism, which in turn created a source of emotional distress, featured within the dialogues of half the participants living with obesity. Using food as a form of avoidance or for coping with other stressors created a secondary stressor as participants then experienced emotional distress over the type and quantity of foods they chose to eat. Participants linked their eating behaviours with the need to alleviate the mental distress that influenced their emotional well-being. They described a cycle of preoccupation with food, subsequent compulsive eating, and loss of control over food intake. This eating cycle triggered further mental distress because of the shame they felt, perpetuating the cycle. It was apparent that many individuals living with obesity perceived their relationship with food to be addictive in nature because of the ability of food to soothe negative emotions they experienced. However, the guilt and shame experienced after these food encounters exacerbated the impact on their psychological and emotional well-being. Although not all participants were able to point out specific aspects of their eating behaviours that would align with the concept of addiction, they could express its connection to their mental well-being. For example: “Again, it’s the mental stress of it all, but I just, it’s honestly … I look at it like an addiction. I just don’t know how to explain it, except that is just how I look at it.”

The connection between emotional eating patterns and subsequent coping behaviours was apparent:

I’m addicted to eating and sweet, and I’m also an emotional eater, and I was very depressed after my back went out and … uh … I ate and ate. I was really addicted to sweets, I really was, I loved to eat.

Participants living with obesity described very specific internal dialogues they had surrounding this coping behaviour. They shared their experiences of preoccupation with food, subsequent compulsive eating, and loss of control over food intake:

I’ll eat supper and then I’ll sit, soon as I’m done eating I’m sitting on the couch or wherever, I’m already thinking oh no what’s in the fridge that I might eat tonight, what am I going to end up eating that I shouldn’t eat. And I’d just eaten, I’m not hungry and, you know a lot of times I’ll eat stuff that I know I don’t even like but it’s just, I almost get like, I feel like a junkie or something I get, anxious and I can’t sit still if I don’t go do it. So I just do it, and then I know I should not have and then, on and on and on.

Some participants shared specific situations resulting in self-described “binge” eating behaviours:

But I mean there’s other times where you just want to pig out. And I find actually when I come back from the doctor I eat more because of the depression and I feel bad about myself. If I’m stressed out I do one of two things, I either don’t eat anything for days or I binge and I eat friggin everything.

This participant identified that visits with her physician could cause her to feel negatively about herself and trigger her depression. In her statement, she linked her stress after visiting her doctor with compensatory eating behaviours to soothe the emotional distress she felt.

All participants who described using food as a coping mechanism admitted great difficulty in trying to overcome their current eating behaviours. A few participants analogized the burden they were experiencing to breaking common addictions:

It’s the same with people trying to stop drinking alcohol or trying to stop smoking. I would even say quitting smoking is easier than stopping eating because you can stop smoking completely. Where you still have to eat something, then you want to eat more and more and more, when do you stop?

One participant believed her eating behaviours affected her mental well-being to such an extent that it should be classified and treated as an eating disorder:

Because I think, my thoughts are, there’s too much anxiety and obsession about food and, all the things that I do that lead to me eating too much I think, it’s an eating disorder. And I don’t think it’s any different than someone who has anorexia and can’t eat because they’re so obsessed with food.

Over half of the HCPs interviewed also likened many of their patients’ eating habits to addictive behaviours that arose as a symptom of their mental well-being issues: “Food cravings for some people. Some of the people that are seeing Mental Health as well, they are dealing with food cravings and kind of try to fill in some psychological need with food.”

Other HCPs actually placed the label of “food addiction” on their clients’ behaviours and acknowledged their need for psychological help:

For some of these people, I think food is an addiction. It is an addiction for them and it is very similar to any other addiction, and therefore they need that piece. I’ve tried to get them, to say, “Why don’t you go and talk to somebody, like even a clinical psychologist and I can refer you.”

It is clear from the quotes above that the use of food as a coping mechanism negatively impacted the mental well-being of those living with obesity. Their feelings of preoccupation with food and subsequent loss of control over intake impaired their environmental mastery. The psychological turmoil experienced from the difficulty in overcoming this behaviour pattern also caused emotional well-being issues as participants expressed emotional distress over their eating habits.

### Interpersonal level

In the interpersonal environment, two themes were identified: (a) *blame and shame by family members and friends because of their weight*, and (b) *condemnation and lack of support from healthcare professionals*. Participants described that both friends and family blamed them for their weight gain, often through both subtle and blatant comments. This was not perceived as deliberately trying to be hurtful, but intensified the blame and guilt they were already feeling for gaining weight:

And when you don’t get the result, you start looking for blame. So, you blame yourself. So, it’s that cycle of blame. And then you have your family who are saying, “Well, what are you doing? What are you doing wrong then? You are not losing any weight. You’re gaining. What is going on?”

Another participant found it especially impactful to her mental well-being when comments were made to her after regaining some weight she had lost: “But, uh, when you gain it back it’s double bad, because they look at you and say did you gain weight, did you gain weight again, so that’s, that puts you down mentally again.”

Similarly, after being notified he had gained “a lot” of weight by a friend he had not seen in a while, this participant explained the impact on his emotions. In his words, he felt: “Horrible. Oh, my god, I just ate more. I had absolutely no control. And I mean it’s part of the reason why I just don’t participate in my community.”

The blame on the individual living with obesity as being in full control of their weight often resulted in them being shamed because of their appearance. Upon reflecting on the positive comments made by family and friends, a participant who had just lost a lot of weight realized what they must have thought of her previous appearance. Her conclusion affected her mental well-being:

My parents, my other friends, they keep complimenting me on how great it is and how great I look. It’s almost … You can just feel that they are almost going to say, “Oh my god, you were so huge, you were ugly.” It’s just almost going to come out there by the number of compliments. It’s like, you know, I was a real person back then too.

The wording in this participant’s statement about being a real person alludes to the fact she felt her family members and friends perceived her previous appearance as less socially acceptable. Her interpretation of them assuming she was ugly at her previous size likely impacted her feelings of social integration and acceptance.

Overall, those living with obesity did not feel they were socially accepted because of their weight, indicated by the blame-filled comments made by family members and friends. This resulted in a decline in their social well-being as well as emotional well-being because the lack of acceptance of their weight resulted in feeling ashamed about their body shape and size.

Like their interactions with their family members and friends, participants’ interactions with HCPs were often framed in the context that weight is completely within their control. Some experienced condemnation for assumed behaviours:

But I didn’t get that from the healthcare system. From them I get stop eating the junk food or you know, the blame, or you’re not exercising enough or whatever else. So, it is frustrating when they are not really listening.

It appears this participant perceived that HCPs are not interested in her personal story and how this has influenced her overall health. There is a sense of strain in communication channels in the clinician–client relationship that resulted in feelings of condemnation.

There was also a sense that HCPs trivialized weight issues when brought forward by patients. In the words of one participant:

There’s such a stigma there that is reinforced. But when I enter the health system, the first place I expect to talk candidly about my issue, I hear the blame. “Well, you know, if you eat right and exercise, you’ll lose weight. It’s as simple as that. It’s a simple thing.” That’s what I get—It’s so simple.

The negative impact on the social well-being of individuals living with obesity was apparent for those who experienced these dialogues of condemnation:

“You’re big. We don’t care.” That is exactly how I feel. I know I shouldn’t. I realize there are a lot of people with a lot of illnesses. But when you’re trying to do something about your weight and that, and you can’t because you can’t walk, you can’t crawl up stairs, you can’t breathe, they just seem to shuffle you from one thing to the other.

From the HCPs’ perspectives, a few described experiences they had witnessed or experiences their clients living with obesity had shared regarding condemnation expressed by other HCPs. A dietitian recalled a story told to her by her client regarding an interaction she had regarding weight management with her family physician:

It was very derogatory. I am trying to remember. “You’ve got to lose weight. You need to eat a lot less. You are not doing enough activity.” Not really listening to what she [the client] is really doing. Like she is doing all of those things, and she is still losing weight but he [the physician] is not giving her any recognition for what she is doing.

Another dietitian also reflected on her coworkers’ responses to one of their patients: “I do think some nurses did judge her by their comments that they would say to me. Like, ‘You didn’t get here by eating healthy.’ Like they had like a lot of snide remarks.”

One clinician recognized that many HCPs believe that weight is completely within our control, resulting in patients living with obesity being blamed for not being able to manage their weight:

Again, I think we as health professionals are influenced to think that the individual is responsible, and somehow they don’t have the willpower or whatnot to choose more wisely with respect to the foods that they eat. And, I think we label them as lazy, and I think we label them as a lot of things.

As in their interactions with family and friends, participants felt condemnation from healthcare professionals because of their weight, resulting in a deterioration in feelings of social acceptance and thus social well-being in the healthcare environment.

### Organizational level

In the organizational environment, one main theme was identified as *ineffective obesity management strategies and the mental well-being supports needed*. About half of the participants thought there were shortcomings in the healthcare system in addressing mental well-being issues in relation to obesity. The participants living with obesity had various experiences in how services related to weight management were delivered. In some cases, participants started with their family doctor and were referred to outpatient or private practice dietitians to assist them in managing their weight, and in other cases they were counselled about losing weight in clinics specializing in chronic diseases related to obesity, such as diabetes. Within this specific province in Canada there is only one obesity management programme with a multi-disciplinary team focused on an evidenced-based approach for long-term weight management. This programme is run by the provincial health authority but requires that clientele pay some out of pocket for the services. No participants in the study noted that they were specifically a part of this programme.

In the participants’ experiences, they noted weight-management programmes often lacked addressing any psychological issues connected to living with obesity, and that no weight-management programme was comprised of a cohesive team that addressed both the physical and mental well-being needs of clients. In the words of one participant: “So, you get lots of how-to stuff without ever addressing some of the psychological stuff.”

Another individual living with obesity pinpointed psychological issues as the cause of being unsuccessful in previous weight-management programmes. She discussed needing to address the aspect of her life that negatively impacted her emotional, psychological, and social well-being before she could maintain any long-term weight loss. In her words:

They do work but for me there are so many other things wrapped up in my weight problems that, that’s not going to last for me until, in my mind—until I deal with some of the reasons I got to this point. And none of those programmes address the mental and the emotional/psychological side of, you people who have maybe 20 or 50 lbs to lose it may just be that they need to learn how to eat properly. But somebody in my position, there is a lot more to it.

When reflecting on her experiences in trying to manage her weight, another participant was left questioning whether she was seeking help and support from the right HCPs:

No, I think it’s really in your mind. And it’s not about the food. I learned that a long time ago—it’s not about the food. It’s about the mind and how it works and how you feed the mind. And I think that if you can feed the mind enough positive, the right decisions will come and the weight will change. That is my bottom line. So does that mean I’ve got to go to Mental Health?

Beyond participants recognizing the need to incorporate support for mental well-being into weight-management programmes, they recognized that current programmes lacked cohesiveness in addressing both physical and mental well-being: “I think I need to be involved in more than one; I guess a programme or something that involves three different things: exercise, nutrition, and psychology. I haven’t found anything that offers all three.”

Similarly, multiple HCPs also recognized a missing component of obesity management was addressing mental well-being: “But I think sometimes there’s other links and parts of the picture, like other team members, mental health especially, that is missing. And if that part sometimes was there, I think you’d see a lot more success.”

Although this participant did not elaborate on why she thought there would be more success if mental health professionals became more involved in obesity management, another practitioner noted the key to greater success was in providing adequate supports that promote mental well-being:

There aren’t supports for people that are trying to lose weight. I don’t know. I mean those linkages aren’t there really in place well anyway with Mental Health. And sometimes I don’t feel like the doctors are really on board. They think it is a food issue and that’s it. So, I don’t feel like the supports are there for those people.

However, a lack of resources to provide mental healthcare within the current system was viewed as a broader issue: “And that psychology piece, that mental health piece is missing in pretty much every aspect of healthcare because they are barely surviving as it is to do the critical stuff let alone, you know.”

At this level, we can consider the lack of mental well-being supports to be a hindrance in providing effective obesity management programmes. Both those living with obesity and HCPs recognized that not only physical but also mental well-being could be compromised for those living with obesity. They also both recognized the role of discussing psychological, emotional, and social issues with mental health experts as an important part of obesity management.

To improve obesity management programmes, we need to know what mental well-being supports individuals with obesity think might be effective in managing their weight. Those living with obesity and HCP provided a glimpse into some things they felt should be included for them to be successful.

The idea of creating a consistent environment of understanding to promote positive mental well-being was an important part of participants’ needs:

Meetings are fine but having that one-on-one with somebody to be able to tell you, you know … we understand. And we get that it’s coming from there, and you know … like they have specific times set aside say they do Monday mornings and anyone who needs to come in to discuss that have a 5-minute time slot and you kind of come.

It is clear from the quote above that this participant desired additional follow-up and guidance from HCPs in the form of regular brief meetings to discuss her success and struggles in relation to weight management with someone she has built a rapport with.

Likewise, one nurse believed that providing an atmosphere of understanding was important when discussing weight with clients living with obesity: “I give them a lot of reassurance that it is not hard to be overweight today, and it’s not your fault, and all that kind of stuff.”

Another key aspect noted to promote positive mental well-being was the creation of a no-judgment zone. Speaking about what made a successful relationship with a HCP, one participant noted: “I think I had to feel comfortable with her, and I think I had to feel un-judged because judgment is what put me there in the first place.”

A dietitian also discussed the importance of remaining free of judgment in her relationships with clients: “They need to know that … I find they need to know that you are there for them. That you are not judging, and that you are there because you care about them.”

Those living with obesity also noted a “we are in this together” approach to managing weight as an important strategy to promote positive mental well-being: “So, I’d really like to see something that covers it all within mental health surrounding it and support wise, an introduction to new things and clarity on what’s available, what’s not available that you’re not alone.”

Similarly, a nurse noted the importance of a positive team-based approach to treatment options so patients feel supported. When speaking with patients, she discusses treatment options by saying: “‘Oh, we’ll just do this. Oh, well, we can do that. We can beat that. We have the answers. It’s not impossible. I can see possibilities here.’”

From the participants’ comments regarding their experiences with weight-management programmes, creating a client-centred approach to care that heavily emphasizes a positive client–practitioner relationship is essential to adequately support the mental well-being of those living with obesity.

### Community level

In the community environment, one major theme was identified: *the negative impact of the social stigma of obesity on mental well-being*. Here, we saw participants identify weight stigma and the resulting weight-based stereotypes expressed to them as being a significant part of their interactions in the community. Weight stigma is negative weight-based attitudes and beliefs that are manifested in stereotypes directed towards individuals because of their weight status (Ratcliffe & Ellison, 2015). Most participants living with obesity were very aware they were stigmatized by the public. As one participant shared:

There is still that social stigma in obesity. Obese people, it’s their own fault because they are lazy and they are stupid and they are just lazy. All they have to do is eat less. That’s what people think. Why don’t you just eat less? I heard that before.

For some the social stigma of their weight was expressed to them through body language and non-verbal communication:

I always feel like there are people looking at me, thinking or saying in their own little head like it’s not that hard to go to a gym. But at the same time, I don’t know, I guess I do feel to some degree that people are looking at me in a negative way.

In other instances, the social stigma resulted in negative verbal confrontations. The statements spoken were often based on stereotypes held about individuals living with obesity. In the words of one participant:

Um, I certainly have experienced strangers coming up to me and insulting me because I was fat, I am fat. So, you know people seem to think that they have the perfect right to call you all kinds of names, and my children are experiencing that as well. So, you know, people think you’re lazy, people think you’re stupid, people think you’re dirty. When they see that you’re fat they think all those things.

Speaking of her interaction with sales clerks at a retail store, one woman noted:

People really treated me … I was almost treated like somebody who is homeless or poor or didn’t have money. There was always some sort of assumption that because I’m big then I must not be important or that I must not have enough money.

The harmful impact of social stigma was evident in the participant stories: “I don’t like being treated like some kind of worthless individual because I have a weight problem.”

Most HCPs did not acknowledge the broader social stigma attached to obesity within the community. However, one participant eloquently discussed the social stigma of obesity:

Unfortunately, I think there’s a big stigma attached to being overweight and obese. I think that we blame the individual for their issues with weight. And we don’t look at the bigger picture and what has led them to that point.

The prevailing social stigma of obesity that exists negatively impacts the social well-being of those living with obesity. Participants shared the hostile relationships they perceived with some people in their communities, making it difficult to feel socially integrated, which negatively impacted their social well-being. As discussed in the next section, it is important to look at the policy level of the environment and how it either contributes to or deters the presence of weight stigma and the resulting negative impacts to mental well-being.

### Policy level

In the policy environment, no major mental well-being theme was identified. This suggests a limited understanding of the impact of factors that operate within the policy level of the SEM. One HCP did discuss the role of policies in the taxation of unhealthy foods and subsidization of healthy foods. None of the participants mentioned the role of policies in ensuring adequate mental well-being support for clients living with obesity. It is important to note this may be due to the fact they were not specifically asked about the role of policies during their interviews. However, there is typically less emphasis on broader societal impacts of obesity in the healthcare system and more focus on individual choice and control.

## Discussion

This study explored the psychological, emotional, and social experiences of individuals living with obesity, along with the perceptions of their well-being from HCP. We identified the most prominent themes that influenced the psychological, emotional, and social well-being of those living with obesity, and categorized them within the SEM. For the individual environment, one main theme was identified, *food as a coping mechanism and source of emotional distress*. In the interpersonal environment, two themes were identified: (a) *blame and shame by family members and friends because of their weight*, and (b) *condemnation and lack of support from healthcare professionals*. In the organizational environment, one main theme was identified, *inadequate support for mental well-being issues in obesity management programmes*. In the community environment, one major theme was identified, *the negative mental well-being impact of the social stigma of obesity*. In the policy environment, no major mental well-being theme was identified.

The use of food as a coping mechanism was an issue many participants dealt with daily. A coping mechanism is a behaviour used to manage internal and external demands of situations appraised as being stressful (Folkman & Moskowitz, 2004). In this case, the use of food to temporarily reduce their distress without attempting to remove or reduce the stressor is considered avoidance coping (Carver & Vargas, 2011). Some participants also referred to their relationship with food as an addiction. Addiction to a substance often includes cravings, loss of control of amount or frequency of use, the compulsion of use, and use despite consequences (Centre for Addiction and Mental Health, 2012). Likewise, HCPs recognized that using food as a coping mechanism was a common experience for many of their clients living with obesity, and providing treatment for this was difficult. HCPs also noted a lack of resources available to address this psychological issue.

The pattern of eating behaviour the participants described has been defined in the literature as a dimension of appetite and intake called hedonic hunger. Hedonic hunger includes frequent thoughts, feelings, and urges about consuming palatable foods in the absence of prolonged food deprivation (Lowe & Butryn, 2007). Although everyone experiences hedonic hunger to some extent, the propensity towards hedonic hunger and hedonic eating varies. A greater propensity for hedonic hunger has been linked to those living with obesity (Lowe & Butryn, 2007). Hedonic hunger in and of itself is a subjective state and must be defined independently of food intake as reports of hunger are not always related to the actual food intake (Lowe & Butryn, 2007). The exact mechanism for this is still up for debate, but includes: (a) the brain reward circuitries in some individuals being dysfunctional, resulting in food becoming hedonically “liked” too much and resulting in overconsumption (Berridge et al., 2010); and (b) the brain reward circuitries becoming passively distorted through overconsumption of palatable foods, triggering overconsumption and compulsive eating of palatable foods (Berridge et al., 2010; Kenny, 2011). Although participants characterized their relationship with food as an addiction, the label of a food addiction is controversial, and the idea of increased propensity for hedonic eating is generally more accepted given that the “liking” and “wanting” of food vary along continuums as well as the psychological cause of the over-eating (Berridge et al., 2010).

Within the interpersonal level of the SEM, blame and shame for their weight status was felt to be expressed indirectly through subtle comments, or through very direct and sometimes harsh comments, as has been previously described (Kirk et al., 2014). The expressions of blame and shame resulted in a deterioration of both the psychological and the emotional well-being of the participants. HCPs that we spoke to did not mention that their patients had told them they experienced blame and shame in their close interpersonal relationships or how this negatively influenced their mental well-being and ability to lose weight.

Participants routinely encountered weight stigma in their day-to-day lives. Previous research supports that individuals living with obesity experience weight stigmatization within their close interpersonal relationships (Puhl, Moss-Racusin, Schwartz, & Brownell, 2008), with family members and friends being the most frequently reported sources of stigma (Puhl & Heuer, 2009). Evaluating the health impact of weight stigma, Lewis and colleagues (2011) confirmed that deterioration in emotional well-being was the most common impact of weight stigmatization for those living with obesity.

Condemnation from HCPs because of excess weight was also a prominent theme within the interpersonal level. This was reported by both individuals living with obesity and HCPs themselves. The pervasive message could be viewed as a lack of care or support for participants living with obesity, which negatively influenced their mental well-being. HCPs acknowledged witnessing weight-biased comments or behaviours from co-workers.

Multiple reviews on weight bias and weight-based discrimination indicate that some HCPs hold stigmatizing attitudes and stereotypical beliefs about people living with obesity (Brown & Flint, 2013; Malterud & Ulriksen, 2011; Puhl & Heuer, 2009). The weight bias of these professionals may negatively affect the quality of care and treatment outcomes for clients living with obesity. Recent research has indicated that some individuals living with obesity who have experienced weight-based discrimination in the healthcare setting report having greater emotional stress associated with contact with medical professionals, a reduction in visits for recommended health screenings, and a reduction in seeking out weight-management assistance (Brown & McClimens, 2012; Phelan et al., 2015; Puhl & Heuer, 2009). Implementation of active training interventions that reduce the weight bias of HCPs is necessary for the physical and mental well-being of those living with obesity (Brown & Flint, 2013).

It is important to note that not all HCPs hold weight-biased attitudes, and there are multiple reasons why they might surface in this population beyond the culture of the healthcare environment itself. An individual may hold weight biases due to family upbringing, the influence of media in the portrayal of those living with obesity, their own personal weight status, personal experiences interacting with those living with obesity, and their interpretation of how their behaviours match or differ from typical stereotypical beliefs (Puhl & Heuer, 2009). The analysis in this research did not identify the cause or extent of weight-biased attitudes and actions those living with obesity felt were expressed by their own HCPs, as these individuals did not participate in the study themselves. This is a limitation as it would have added a depth of understanding to the interactions experienced. It is also noteworthy that national obesity organizations whose goals are to holistically support those living with obesity through initiatives such as reducing weight bias and stigma through research and education have thousands of HCP members (Canadian Obesity Network, 2017). This research is not an attempt to ignore the exceptional efforts of all those HCPs working to support those living with obesity, but we hope it will encourage additional work in reducing weight bias and stigma in healthcare.

Within the organizational level of the SEM, participants identified a lack of psychological support in current obesity management strategies. Individuals living with obesity noted they had psychological issues they needed to address before they could successfully implement the lifestyle changes needed for weight loss. Participants wanted a programme free of judgment, focused on understanding everyone’s struggles, and based on a collaborative approach. HCPs also indicated mental well-being as the missing link to obesity management. They believed this area of healthcare was underfunded and underutilized.

Recent research has indicated that promoting psychological well-being for those living with obesity is beneficial for improving quality of life and promoting long-term weight loss (Presti, Lai, Hildebrandt, & Loeb, 2010; Yilmaz, Povey, & Dalgliesh, 2011). Creating a judgment-free zone in weight-management clinics is crucial for building trust and improving the quality of care patients receive (Gudzune, Bennett, Cooper, & Bleich, 2014). To reduce judgment, educational interventions, focused on reducing the stigma of obesity, are needed for HCPs (Brown & Flint, 2013). HCPs focused on building trust and providing support to clients living with obesity improve the likelihood their clients will achieve their goals (Intarakamhang & Intarakamhang, 2015). Obesity management teams have recognized the value in providing a consistently encouraging office environment, so clients receive the support necessary to feel HCPs are invested in their success (Campos, 2014).

Within the community level of the SEM, the social stigma of obesity was experienced daily by participants through both verbal and non-verbal means. For example, some participants expressed a feeling of judgment arising from the way a person might look at them, while others were subjected to verbal insults that focused on their weight status. Some examples of the negative stereotypes participants felt were directed at them included that they were lazy, stupid, dirty, or poor. The mental well-being implications of these encounters were obvious: a deterioration of both emotional and social well-being. Only one clinician discussed the stigma of obesity in the broader community and its impact on mental well-being. Most HCPs only acknowledged that their relationships with clients had the ability to influence their success.

Research has identified that the community is an environment where weight stigma flourishes. Verbal expressions of stereotypes based on weight are reported to occur predominantly with strangers in the community (Lewis et al., 2011; Thomas, Hyde, Karunaratne, Herbert, & Komesaroff, 2008). Furthermore, current research on the cyclic obesity/weight-based stigma model suggests that weight-based stigma results in psychological stress, encouraging emotional eating, which results in weight gain, heightening the weight-based stigma experienced (Tomiyama, 2014). This model reveals the complex interactions of the different environments that work against the mental well-being of those living with obesity.

In this study, participants did not identify the role of policies in influencing their mental well-being in relation to living with obesity. Although one HCP discussed policy in relation to the taxation of unhealthy foods and healthy food subsidies, they did not identify specific policies in relation to weight bias or the mental well-being of those living with obesity. This highlights the importance of increasing awareness of weight bias and weight-based stigma in the public and among HCPs to increase support and uptake of policies focused on improving the mental well-being of those living with obesity. Such policies would include adding weight to all provincial as well as federal human rights codes so that service providers and employers are unable to discriminate against someone because of their body shape or size.

Public health policies require a shift away from strategies focused on the paradigm of individual choice, to strategies focused on the broader social effects of obesity such as weight-based stigma and bias (Johnston, Matteson, & Finegood, 2014). However, a population-level policy approach to obesity management requires advocacy and support from the public to encourage government implementation (Huang et al., 2015; Lange & Faulkner, 2012). The public’s classification of obesity as caused by individual behaviour, or attributed mainly to environmental factors outside one’s control, influences the amount of public support for policies that either penalize or assist individuals living with obesity (Thibodeau, Perko, & Flusberg, 2015). Raine and colleagues (1989) found that key Canadian influencers of public health policy in government, workplaces, school boards, and print media strongly endorsed (99%) individual-focused obesity policy approaches. A survey of undergraduate students at the University of Toronto revealed over 50% support for redistributive, compensatory, and price-raising policies as part of obesity management (Lange & Faulkner, 2012). Of the few Canadian studies that looked at potential obesity-related policies, most focused on individual behaviour change and environmental strategies. Only one anti-discrimination policy focused on weight bias was even suggested as a potential strategy (Lange & Faulkner, 2012). Little research has addressed the need for obesity-related policies targeting improvements in mental well-being. This may suggest that the public does not see the connection between physical and mental well-being issues, and therefore does not demand solutions to address these issues.

There are several strengths to this study. To our knowledge, we are the first to examine the mental well-being experiences of those living with obesity across levels of the SEM, and the perceptions that HCPs have of the mental well-being issues experienced by their clients living with obesity. Exploring mental well-being outcomes in relation to different environmental levels broadened our understanding of how complex daily interactions within the environment can have a very significant impact on the mental well-being of those living with obesity. Furthermore, this analysis provided evidence of the need for mental well-being supports across all environmental levels of the SEM. Using the SEM added depth to the analysis as it highlighted that psychological, emotional, and social issues arising as a response to personal weight status surfaces in multiple aspects of the lives of those living with obesity. Having found negative impacts to psychological, emotional, and social well-being within four different environment levels of the SEM, the study emphasizes the profound influence of weight bias on the mental well-being of those living with obesity that may not have been exposed without using this framework.

It was also important to include the perceptions of HCPs because their understanding of obesity-specific influences on quality of life and successful weight management will indicate whether they can develop realistic weight-management plans that will support the psychological, emotional, and social well-being of their clients (Vallis, 2016).

The limitations of this study include the use of secondary data from interviews where the original questions posed to participants focused more broadly on the perceptions and experiences individuals living with obesity had about their weight, as well as how HCPs, when assisting with weight management, perceived interactions with individuals living with obesity and the healthcare system (Kirk et al., 2014). Mental health issues were therefore not the focus but emerged during analysis of the narratives of both groups in the original study. Because there were no specific probes around the dimensions of emotional, psychological, and social well-being at the time the interviews were conducted, these dimensions were only discussed in relation to negative experiences and the deterioration of mental well-being. It is important to note that had specific questions been asked about participants’ mental well-being, participants’ responses may have also included more positive aspects of mental well-being in relation to living with obesity.

Additionally, the research methods, including sample size, limit the ability to generalize outside the eastern Canadian province where the interviews were conducted. The participants in this study were also predominantly female and may have had different mental well-being experiences in relation to living with obesity compared to males, if interviewed, due to many factors such as societal norms regarding the social acceptability of excess weight in each gender. Furthermore, the original data were collected in 2010–2011, making them already several years old.

We recognize these limitations and strived to stay close to the data, discussing themes that arose clearly from participant dialogues rather than arising through our own assumptions. We also tried to remain sensitive about our unavoidable preconceptions by reporting opposing views and through consideration of the research limitations.

Despite these limitations, given the paucity of information available on the concept of mental well-being within obesity management, it appears there has been minimal, if any, progress in this regard since the original data were collected. Whereas the original study on which this analysis was based applied a feminist postructural methodology across multiple levels of the SEM, applying a mental well-being framework enabled us to more fully elucidate aspects of mental well-being within participant narratives, and allowed additional themes specific to mental well-being to be further explicated. The rich dialogue of our participants provides direction for future research to understand social and environmental root causes and consequences of the mental well-being issues experienced by individuals living with obesity.

## Conclusions

Individuals living with obesity may face negative mental well-being impacts because of their weight status in multiple levels of their environment. Based on our findings, these were the use of food as a coping mechanism and source of mental distress; blame and shame in interpersonal relationships; condemnation and lack of psycho-social care within the healthcare system; and the negative impact of the social stigma of obesity. Weight bias directed at those living with obesity was an overarching theme and main source of the negative interactions that caused deterioration of the participants’ mental well-being, as we have found in our previous studies (Aston et al., 2011; Kirk et al., 2014; Price et al., 2015). While HCPs could identify the individual- and organizational-level issues as barriers to promoting the positive mental well-being of their clients, at times they appeared to lack awareness of the social determinants of health or the need to look beyond the individualistic healthcare model to provide support for their clients.

These data should encourage health professionals and policymakers to re-evaluate obesity management strategies and obesity policies to better address mental well-being. Until we recognize and make a significant effort to reduce weight bias in healthcare organizations and society alike, we will be unable to adequately provide the mental well-being support needed for those living with obesity. Researchers can use the insight provided in this study to develop obesity management programmes and policies concentrated on environmental changes that actively promote positive mental well-being.
